# Unanticipated critical findings on echocardiography in septic patients

**DOI:** 10.1186/s13089-020-00162-x

**Published:** 2020-04-02

**Authors:** Sarah J. Beesley, Ezekiel Egan, Michael J. Lanspa, Emily L. Wilson, Elliotte L. Hirshberg, Colin K. Grissom, Rebecca Burk, Samuel M. Brown

**Affiliations:** 1 Pulmonary Division, Intermountain Medical Center, Salt Lake City, UT USA; 2 Pulmonary Division, University of Utah School of Medicine, Salt Lake City, UT USA; 3 University of Utah School of Medicine, Salt Lake City, UT USA; 4 Critical Care Echocardiography Service, Intermountain Medical Center, Salt Lake City, UT USA; 5 Department of Pediatrics, University of Utah School of Medicine, Salt Lake City, UT USA; 6 Shock Trauma Intensive Care Unit, 5121 South Cottonwood Street, Murray, UT 84107 USA

**Keywords:** Echocardiography, Intensive care, Sepsis, Septic shock

## Abstract

**Background:**

Echocardiography is increasingly performed among septic patients as a routine part of evaluation and management in the intensive care unit (ICU). The rate of unanticipated critical findings (e.g., severe left or right ventricular dysfunction or pericardial tamponade) on such echocardiograms is unknown. We evaluated a retrospective cohort of septic ICU patients in whom transthoracic echocardiography was performed as a routine part of sepsis management. In addition to identifying critical findings, we defined whether each critical finding was anticipated, and whether the clinical team responded to the critical finding. The primary outcome was rate of unanticipated critical findings, which we hypothesized would occur in fewer than 5% of patients. We also performed an exploratory analysis of the association between unanticipated critical finding and mortality, controlling for severity of illness.

**Results:**

We studied 393 patients. Unanticipated critical findings were identified in 5% (95% CI 3–7%) of patients (*n* = 20). Among the 20 patients with unanticipated critical findings, a response to the unanticipated critical finding was identified in 12 (60%) patients. An unanticipated critical finding was not significantly associated with 28-day mortality when controlling for admission APACHE II (*p* = 0.27).

**Conclusions:**

Unanticipated critical findings on echocardiograms in septic ICU patients are uncommon. The potential therapeutic relevance of echocardiography to sepsis is more likely related to hemodynamic management than to traditional cardiac diagnoses. Research studies that employ blinded echocardiograms in septic patients may anticipate unblinding for critical findings approximately 1 in every 20 echocardiograms.

## Background

Echocardiography is increasingly performed among patients with sepsis as a routine part of management [1–3]. Opinion and/or consensus statements in favor of broader application of focused critical care echocardiography and instructional guides [4–8] manifest increasing momentum in favor of the broad use of these focused echocardiograms. Critical care echocardiography now supports a board certification process [9–11].

Appropriateness criteria suggest that transthoracic echocardiogram (TTE) is appropriate in the case of shock or situations where TTE is likely to change management, and a variety of studies suggest that TTE identifies findings that change management [12–18]. Admittedly, these studies have either been in populations with high likelihood of relevant findings (e.g., perioperative management of cardiac surgery patients) or have been analyzed under the assumption that echocardiography should direct management and then reporting that echocardiography changes management [19]. Currently, the application of echocardiography in septic patients is often related to hemodynamic management, especially fluid administration [20–22].

However, for healthcare systems evaluating the implications of screening echocardiograms and for clinical researchers wondering how often a research echocardiogram may identify an unanticipated critical finding (such as severe left or right ventricular dysfunction or pericardial tamponade, which may necessitate new study procedures and/or unblinding), it is important to know how often an echocardiogram obtained for sepsis identifies an unexpected critical finding. In a prior study of an unselected ICU population (*N* = 467), a high proportion (36%) of unanticipated cardiac abnormalities were identified on echocardiogram [23]. This study included many findings not normally considered critical and was not specific to sepsis patients.

We thus sought to clarify how often unanticipated critical findings are identified in the echocardiograms of septic patients admitted to the ICU. We performed an analysis of patients in our registry of septic patients in whom TTE had been performed as a routine part of the management of sepsis. We hypothesized that unanticipated critical findings on echocardiogram would be present in less than 5% of echocardiograms.

## Methods

### Patients

Participants included patients (age at least 18 years) with severe sepsis or septic shock (Sepsis-2 definition [24], which applied at the time of cohort assembly) admitted to study ICUs between October 2012 and November 2015. Patients had clinically suspected infection defined by study coordinator chart review, two or more systemic inflammatory response syndrome criteria and had either shock (systolic blood pressure (SBP) < 90 mmHg despite IVF challenge of ≥ 20 mL/kg or infusion of vasopressors) or severe sepsis (lactate > 4 mmol/L) [24, 25]. We included only patients who had an echocardiogram obtained within 24 h of admission; in the study ICU, this is approximately 50% of patients with sepsis and 75% of patients with septic shock (unpublished data). Exclusion criteria included known pregnancy, primary diagnosis of acute coronary syndrome or major cardiac dysrhythmia, or known alternative diagnosis for shock (trauma, anaphylaxis, hemorrhage) on coordinator review, confirmed by an investigator.

### Critical findings on echocardiogram

We reviewed all echocardiogram reports to identify critical findings. Echocardiograms were performed by trained, credentialed sonographers and over-read by either board-certified cardiologists or Level II critical care echocardiographers who were testamurs of the National Board of Echocardiography ASCeXAM. We employed the American Society of Echocardiography definitions of critical findings: (1) cardiac tamponade or new large pericardial effusion, (2) new left ventricular ejection fraction (LVEF) < 30%, (3) new severe right ventricular systolic dysfunction [26], (4) evidence of aortic dissection, (5) new cardiac masses or thrombi, or (6) new, severe valvular dysfunction [27]. We also included (7) left ventricular outflow obstruction or hypertrophic cardiomyopathy with velocities > 4 m/s, or (8) moderate or greater right to left shunt [28], as those may be immediately relevant in a critical care environment.

**Fig. 1:**
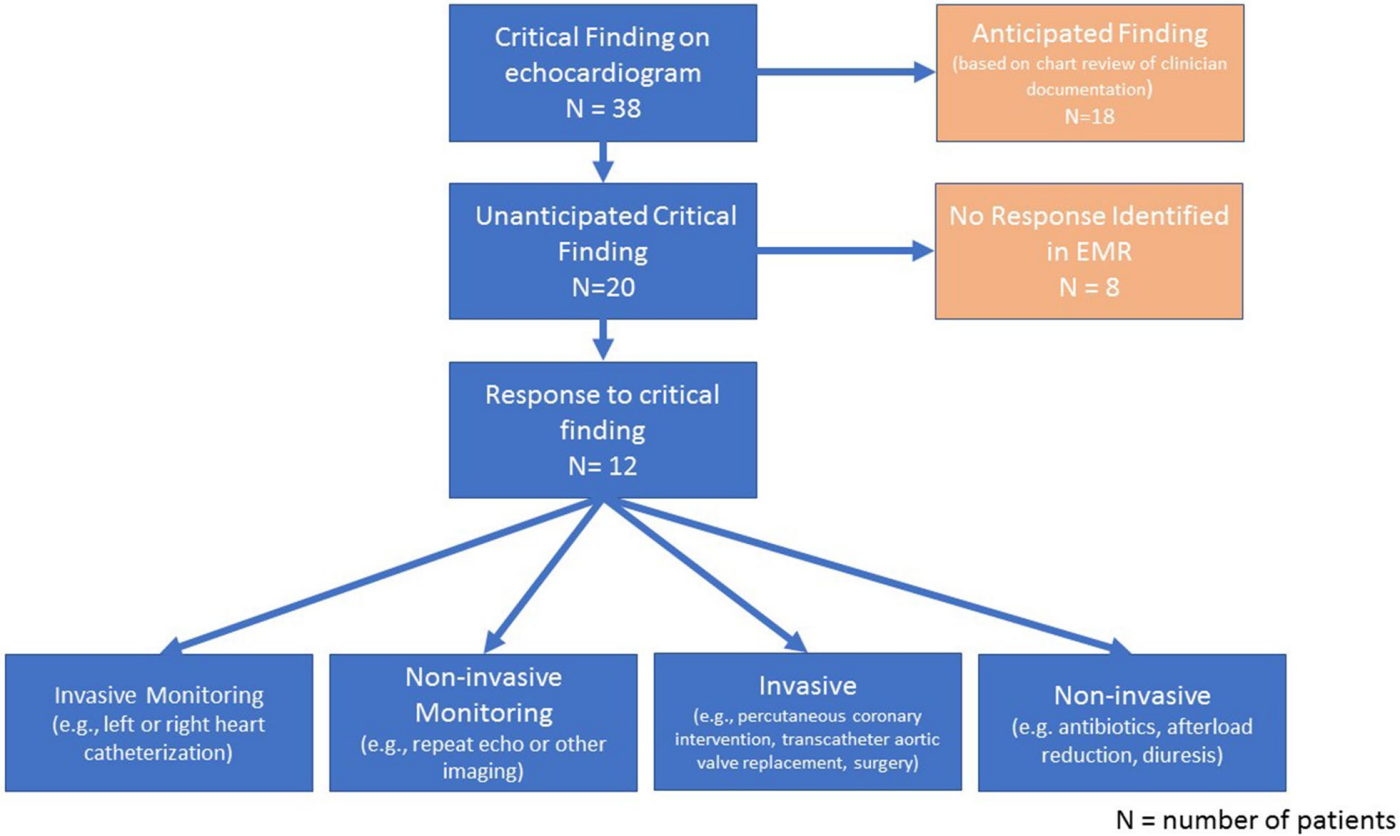
Study flowchart

We defined whether the critical finding was anticipated or not based on the following algorithm: (1) was the finding present on a prior echocardiogram? (if so ⟶ anticipated); or (2) was the finding referenced in clinical documentation as being of diagnostic concern before the echocardiogram had been performed (if so ⟶ anticipated). See process outline in Fig. 1. Of note, when a vegetation was identified on TTE, this was not considered an unanticipated critical finding if endocarditis was considered prior to obtaining the TTE; we considered positive blood cultures with a typical organism before the echocardiogram was ordered as suggesting that endocarditis was under diagnostic consideration.

We also evaluated the response to the identification of an unanticipated critical finding. If the finding was unanticipated, the clinical response was noted in the following categories: no response, monitoring response, or intervention. No response means that we could find no evidence in the electronic medical record (EMR) that the treating team incorporated the critical finding into their monitoring or management. Monitoring response means that the EMR indicated either increased or additional monitoring explicitly tied to the critical finding. This included both non-invasive monitoring (e.g., repeat echocardiogram or other imaging), and invasive monitoring (e.g., left or right heart catheterization). Intervention was considered present if there was a change in treatment associated with the critical finding and included both non-invasive intervention (e.g., antibiotics, afterload reduction, diuresis) and invasive intervention (e.g., percutaneous coronary intervention, transcatheter aortic valve replacement, surgery).

A critical finding was designated as a false-positive finding if subsequent testing refuted the apparent findings from the initial echocardiogram report.

### Analysis plan

We estimated a priori that the proportion of unanticipated critical findings would be < 5%. We estimated the precision of our estimate to be within 2.3% for a proportion of 5% with a sample size of 390 and a 95% confidence level. This was calculated using PASS 12.0.3 [29].

The prespecified primary analysis was to estimate the proportion of unanticipated critical findings identified on TTE. As a secondary exploratory analysis, we evaluated the association between critical findings and 28-day mortality, adjusted for severity of illness, using multivariate logistic regression. All analyses were conducted using the R Statistical Package, version 3.5.1 [30].

The study was approved with waiver of informed consent by the Intermountain Institutional Review Board (Intermountain IRB #1009957).

## Results

This cohort of septic patients included 393 patients. There were 222 (56%) patients in shock (systolic blood pressure < 90 mmHg) at the time of the echocardiogram; 151 of these were on vasopressors. Mean APACHE II score for the cohort was 26 (SD = 10). Overall 28-day mortality was 24% (*n* = 93).

Unanticipated critical findings were identified in 5% (95% CI 3–7%) of patients (*n* = 20). In total, 40 critical findings were identified on TTE in 38 (10%) patients (two patients had two critical findings). Of these 40 findings, 21 (53%) were unanticipated (see Table 1). Twelve of the 19 anticipated critical findings had been identified on prior echo, and the remaining 7 anticipated critical findings were previously suspected by the treating team based on EMR documentation.

**Table 1:** Critical findings

Type of critical finding	Critical finding (*N* = 40)	Unanticipated critical finding (*N* = 21)
Tamponade	0 (0%)	0 (0%)
New depressed LV EF < 30%	9 (22%)	7 (33%)
New severe right ventricular systolic dysfunction	5 (12%)	2 (9%)
Aortic dissection	1 (2%)	1 (5%)
Cardiac mass, thrombus or vegetation	13 (32%)	7 (33%)
LVOT obstruction or HCM, > 4 m/s	3 (7%)	2 (9%)
Right to left shunt, moderate or severe	1 (2%)	1 (5%)
Severe valvular dysfunction	8 (20%)	1 (5%)

Data presented as *N* (%); *N* represents number of critical findings (there are two patients with two critical findings)

*LVEF* left ventricular ejection fraction, *LVOT* left ventricular outflow track, *HCM* hypertrophic cardiomyopathy

Out of the 20 patients with unanticipated critical findings, a response to the unanticipated critical finding was identified in 12 (60%) patients. The response was non-invasive monitoring in 11 patients (primarily repeat echocardiograms) and non-invasive intervention in 6 patients (primarily blood pressure or fluid management). Some patients had multiple responses to the unanticipated critical findings, i.e., both a change in monitoring and a non-invasive intervention. No patients with unanticipated critical findings underwent either invasive monitoring or invasive interventions for that finding. Four unanticipated critical findings (one aortic dissection and three intracardiac masses) were found to be false-positives on repeat imaging with either CT angiogram or echocardiogram. In the 8 patients whose critical finding elicited no response, 50% died within 48 h of ICU admission; among the 12 patients whose critical findings elicited a response, 1 (8%) died within 48 h of ICU admission.

An unanticipated critical finding was not significantly associated with 28-day mortality when adjusted for admission APACHE II (*p* = 0.27). A critical finding (whether anticipated or unanticipated) on echocardiogram was also not significantly associated with 28-day mortality when adjusted for admission APACHE II score (*p* = 0.85).

## Discussion

As echocardiograms are frequently performed both clinically and within research protocols, we sought to identify how often the results of an echocardiogram performed among septic ICU patients were unexpected and critical. In our large single-center cohort that makes extensive use of echocardiography in the early management of septic patients, unanticipated critical findings were identified in 5% of septic patients. This confirmed our prespecified hypothesis before this study that approximately 1 in 20 echocardiograms in septic patients identified an unanticipated critical finding. Mortality in this study is comparable to other studies on similar populations [31, 32].

Our findings provide useful estimates for researchers performing blinded echocardiograms in septic patients of the probability that unblinding may be required. Our findings also suggest to clinicians how often unexpected results may be anticipated on echocardiograms performed as part of the management of sepsis. We also believe that our findings suggest that the key target for building evidence for efficacy of critical care echocardiography in septic patients will emphasize hemodynamic guidance, which is likely to be much more important than unanticipated diagnoses.

Our findings contribute to preexisting literature on critical care echocardiography. In a study of pediatric ICU patients, echocardiography introduced a new diagnosis unrelated to the echo indication in 13%; an echo ordered as stat was more likely to change management and diagnosis than a routinely ordered echo [33]. In another study of critically ill patients, TTE changed diagnosis in 19% of cases and management in 34% of cases with “adequate clinical data”. TTE changed diagnosis in 56% of cases and management in 58% of cases with “inadequate clinical data” [34]. For comparison, patients presenting to ED with chest pain and shortness of breath, TTE changed diagnosis in 25% of cases and management in 37% of cases. It also increased the diagnosing physician’s confidence in diagnosis and management [35]. For comparison, rates of unanticipated findings in outpatients undergoing echocardiography for dyspnea or chest pain were found to be 22% in one study of 368 outpatient TTEs [36]. These comparable studies reflect a variety of settings and methods, but with the similar conclusion that, occasionally, unanticipated findings that change management may be identified by echocardiogram.

Our study findings may not be fully generalizable because they represent patients treated at one academic referral center and was performed on a retrospective cohort. Many emergency room physicians at this center have an interest in point of care ultrasound and patients are also often frequently ultra-sounded at the bedside while in the emergency room. This could have potentially led to an underestimation of unanticipated critical findings compared to other sites if findings were identified prior to ICU transfer and documented in the medical chart. We also note the contextuality of our findings: the rate of unanticipated findings on echo may correlate with the breadth of clinicians’ differential diagnoses. At this academic referral center, ICU patients are generally cared for by a team of clinicians prior to ICU transfer, as well as an ICU resident, fellow and attending once in the ICU; having several different clinicians involved in creating and documenting a differential diagnosis may make this less of an issue, although we did not formally address this question. In addition, while a TTE is generally obtained in septic patients admitted to the study ICU, if sicker patients at higher risk of an unanticipated critical finding preferentially underwent echocardiography, our estimate of the prevalence of such findings may be inaccurately high. We did not perform a bubble study on all patients, so the rate of right to left shunt in this population might have been underestimated. We acknowledge that our study is not adequately powered to determine whether unanticipated critical findings—or responses to them—are associated with mortality; much larger cohorts would be required for such an analysis.

## Conclusion

Unanticipated critical findings on routine echocardiograms in septic ICU patients are uncommon. The potential relevance of echocardiography to sepsis management is more likely related to hemodynamic management than to traditional cardiologic diagnoses. Secondarily, research studies that employ blinded echocardiograms in septic patients may anticipate unblinding of approximately 1 in every 20 echocardiograms based on the unanticipated critical findings.

## Data Availability

The datasets generated and/or analyzed during the current study are not publicly available due to lack of consent from participants regarding data sharing.

## Abbreviations

APACHE: Acute Physiology and Chronic Health Evaluation
CT: Computed tomography
CI: Confidence interval
EMR: Electronic medical record
IRB: Institutional Review Board
ICU: Intensive care unit
IVF: Intravenous fluid
LVEF: Left ventricular ejection fraction
SD: Standard deviation
SBP: Systolic blood pressure
TTE: Transthoracic echocardiogram
